# T‐Shaped Remote Underwater Video (RUV): A Cost Friendly and Easy Use RUV Technology to Estimate Fish Length in Small Alpine Streams

**DOI:** 10.1002/ece3.72144

**Published:** 2025-09-08

**Authors:** Paolo Cappa, Matt J. Nimbs

**Affiliations:** ^1^ National Marine Science Centre Southern Cross University Coffs Harbour New South Wales Australia

**Keywords:** Alps, ecological monitoring, freshwater habitat, non‐invasive monitoring

## Abstract

Alpine streams represent some of the most challenging yet ecologically valuable freshwater environments to study, due to their remoteness, fast flows and extreme climatic conditions. Traditional fish survey methods are often impractical or invasive in these habitats. This study presents a lightweight, low‐cost, T‐shaped remote underwater video (RUV) system optimized for fish monitoring in small, high‐altitude streams of the European Alps. Field tests were conducted across three alpine streams in northwestern Italy in June 2025. The RUV system proved effective in recording salmonid presence, estimating fish length using an integrated reference scale, and capturing natural behaviours such as feeding. The compact steel frame (~1 kg) ensured stability in fast‐flowing waters while remaining portable for deployment in remote areas. Despite challenges such as bubble interference and site accessibility, the RUV allowed reliable, permanently recorded observations with minimal disturbance. This method offers a promising non‐invasive approach for ecological research and conservation monitoring in alpine freshwater systems, with potential applications in species distribution studies and behavioral ecology.

## Graphical abstract

This study presents a lightweight and inexpensive remote underwater video (RUV) system designed for monitoring fish in high‐altitude alpine streams. Field tests conducted in the European Alps have demonstrated the RUV's effectiveness in detecting salmonids, estimating their length and recording natural behaviour with minimal disturbance. The method offers a practical, economic and non‐invasive tool for ecological research in challenging aquatic environments.

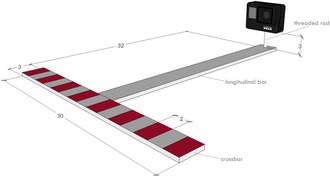

## 1 Introduction

Alpine environments are characterized by high altitude and extreme climatic conditions. These regions are defined as areas of rugged mountain terrain, permanent or semi‐permanent snowfields, tundra, glacial formations and a dynamic network of aquatic habitats (DG‐ENV and Sundseth 2009), including alpine streams, which are narrow, fast‐flowing watercourses that descend down the slopes of mountain ranges (Ward 1994). Typically small in size, these streams are environmentally heterogeneous due to high variation in hydrologic sources, such as glacier melt, snowmelt, groundwater spring, or lake fed (Hotaling et al. 2017). However, this habitat mosaic makes alpine streams home to species that are uniquely adapted to long winters and frigid conditions (Ward 1994).

Studying alpine ecology is key to understanding and conserving the biodiversity of these delicate habitats that are threatened by ongoing climate change (Hotaling et al. 2017) and by past fish stocking of exotic species (Polgar et al. 2022). Factors that are also contributing to the global ‘Freshwater Biodiversity Crisis’ (Harrison et al. 2018; Albert et al. 2021). Yet access to these streams for scientific purposes is often difficult due to remoteness and the topographically complex nature of alpine terrain. Alpine streams can be remote (Hotaling et al. 2017), predominantly located in areas inaccessible to vehicles, within steep‐sided valleys that are difficult to access with bulky equipment. Reaching these streams often means researchers need to undertake long hikes along steep and rocky trails on foot, carrying all their equipment. This logistical challenge limits the use of conventional sampling techniques, such as large and bulky nets and heavy electrofishing units. As a result observing fish behaviour, recording diversity and estimating size in alpine streams presents considerable logistical and methodological challenges.

Traditional fish survey methods, such as electrofishing (Bohlin et al. 1989) or the use of nets (Portt et al. 2006), are logistically impractical in these environments and can also be harmful to fish if performed incorrectly (Snyder 2003). Alternatively, remote underwater video (RUV) systems offer a non‐invasive alternative (Tweedie et al. 2023).

Existing underwater video methods have been widely used in marine systems (Leonetti et al. 2024). However, little research has been conducted using underwater video in freshwater systems, and of the few studies that have, they have been in medium‐sized and substantial streams (e.g., Davis et al. 2017; Coleman et al. 2023; Tweedie et al. 2023). There are no published studies that have used RUV in the European Alps, where logistic constraints due to topography make the majority of commercially available configurations too unwieldy to use in small, fast‐flowing alpine streams; in addition, these set‐ups can be prohibitively expensive. Innovative, lightweight and compact solutions are therefore essential for effective research in these logistically challenging and understudied ecosystems. This pilot study presents the outcome of field trials using a low‐cost, lightweight, compact, single‐camera RUV system optimized for use in small alpine streams, and provides details of its use, with the ultimate goal of providing a reliable tool for data collection in potentially threatened freshwater ecosystems and, consequently, useful information for robust ecosystem management.

## 2 Materials and Methods

Tests were conducted from 23 to 29 June 2025 in three different alpine streams to trial the RUV in a range of environmental conditions, such as variable ambient light level, substrate type, water depth and stream width. Specifically, observations were conducted in *Varaita di Sustra* (44.668085° N, 7.008482° E), *Rio Bulè* (44.652340° N, 7.166481° E) and *Rio Alpetto* (44.672065° N, 7.181239° E), all located in the Cottian Alps in the southwest Piedmont region, Italy. Observations in *Varaita di Sustra* (width ~2.5 m, depth ~0.54 m) were conducted at an altitude of 2087 ± 7 m (SD) within a sub‐alpine level. *Rio Bulè* (1515 ± 12 m; width ~4.5 m, depth ~0.68 m) is mainly located in a high grass meadow, with the latter site overlapping a fragmented tree line, considered located at the upper edge of montane level. Conditions at these locations were characterized by clear water and a substrate composed mainly of rocks and cobbles. In contrast, observations in *Rio Alpetto* (1265 ± 1 m; width ~1.7 m, depth ~30 cm) were carried out within a forested area in the montane level, where limited light penetration and increased turbidity were observed. Here, fine sediment and organic particles became suspended during equipment deployment.

RUVs were positioned at various points along the watercourses, randomly selected, and the utmost care was taken to ensure deployment accuracy (i.e., stable position and upstream direction of the camera) and minimize noise and unnecessary movement to reduce disturbance to fish during installation. Once in the water, RUVs were left to record in a fixed position for 20 min, as this time was considered sufficient and to maximize the number of daily deployments (Tweedie et al. 2023). Researchers moved away from the deployment point more than ~100 m to avoid further disturbance. Upon recovery, RUVs were found in the same position in which they had been deployed, and no sediment scour due to water flow was recorded during the tests, despite the fast‐moving waters.

### 2.1 T‐Shaped RUV

**FIGURE 1:**
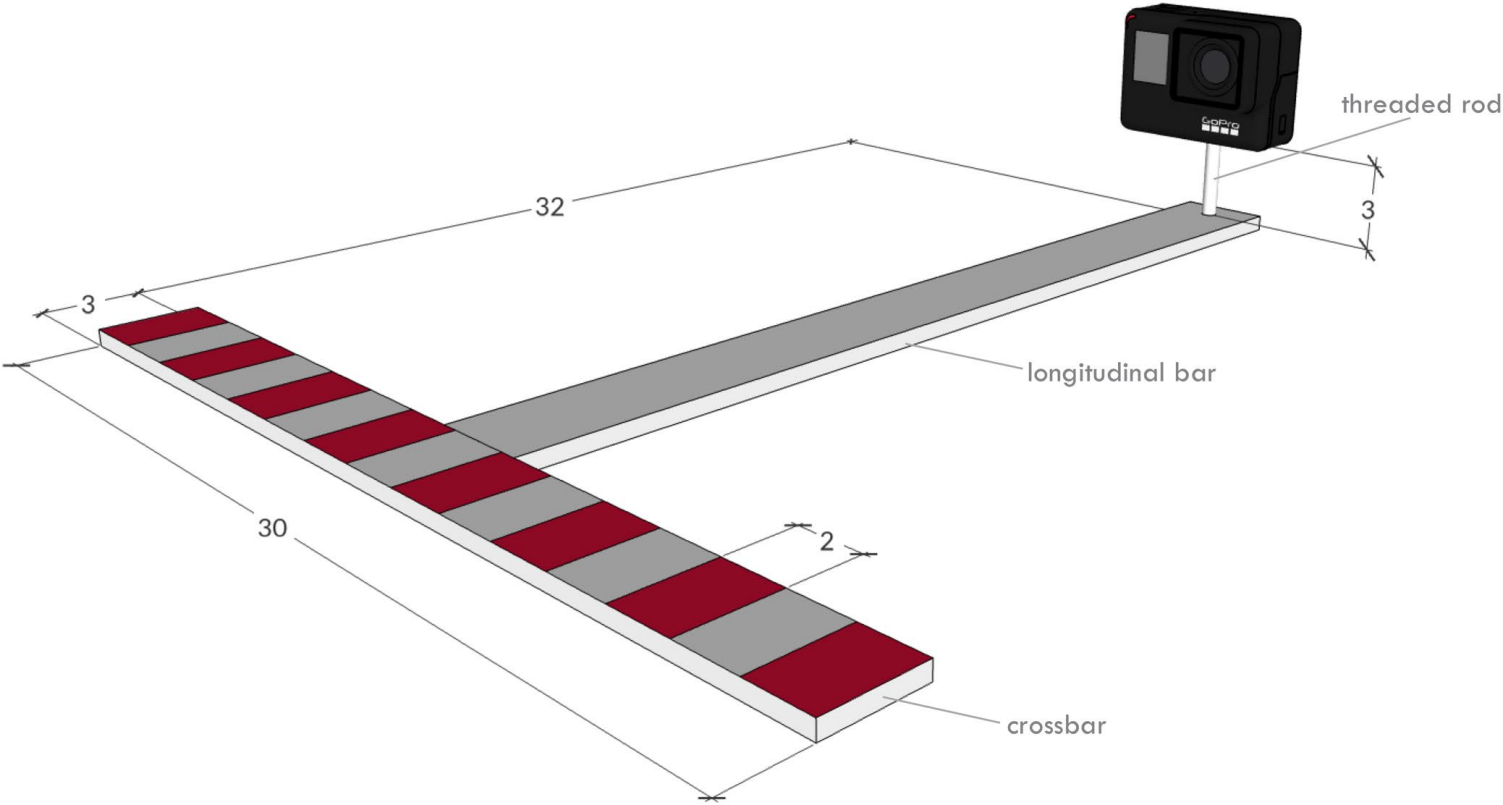
3D view of the T‐shaped remote underwater video (RUV) frame used in this study. The 3D model was created with SketchUp (www.sketchup.com) (units are cm).

The system, hereafter named a ‘T‐shaped RUV’ (Figure 1), consists of a steel frame welded at the central junction, with a crossbar measuring 30 × 3 cm and a longitudinal bar measuring 32 × 3 cm, both with a thickness of 0.5 cm. The frame was initially painted with matt grey paint to improve camouflage with the surrounding environment. A measuring scale, using 2 cm increments, was then red painted on the crossbar for subsequent video analysis. An underwater camera was mounted at the end of the longitudinal bar on a 3 cm high threaded rod, which allows for small adjustments to the camera angle, using the standard mounting bracket supplied with the camera. The camera was positioned to clearly capture the measuring scale at the base of the image. The total weight of the system (excluding the camera) is 994 g.

Field trials were conducted with 3 frames. One T‐shaped frame was equipped with a GoPro Hero 12 Black and the remaining two frames with 2 GoPro 7 White. All cameras used 128GB SD cards. For the GoPro Hero 12 Black, the manufacturer's 1720 mAh battery provides ~70 min of video recording time in 5.3 K and 30 fps format, whereas for the GoPro 7 White, the 1220 mAh battery provided ~60 min of video recording time in 1440p and 30 fps format in the environmental conditions used in this study (water temperature ~10°C); however, a lower water temperature may decrease the battery output.

In the current market (June 2025) the specified cameras are ~€250 for the GoPro Hero 12 Black and ~€100 for the GoPro 7 White, second‐hand. Each SD card was ~€12, and the T‐shaped metal frame cost ~€25. Complete, each system provides a functional, lightweight and inexpensive alternative to more expensive, larger and cumbersome RUV units.

### 2.2 Video Analysis

**FIGURE 2:**
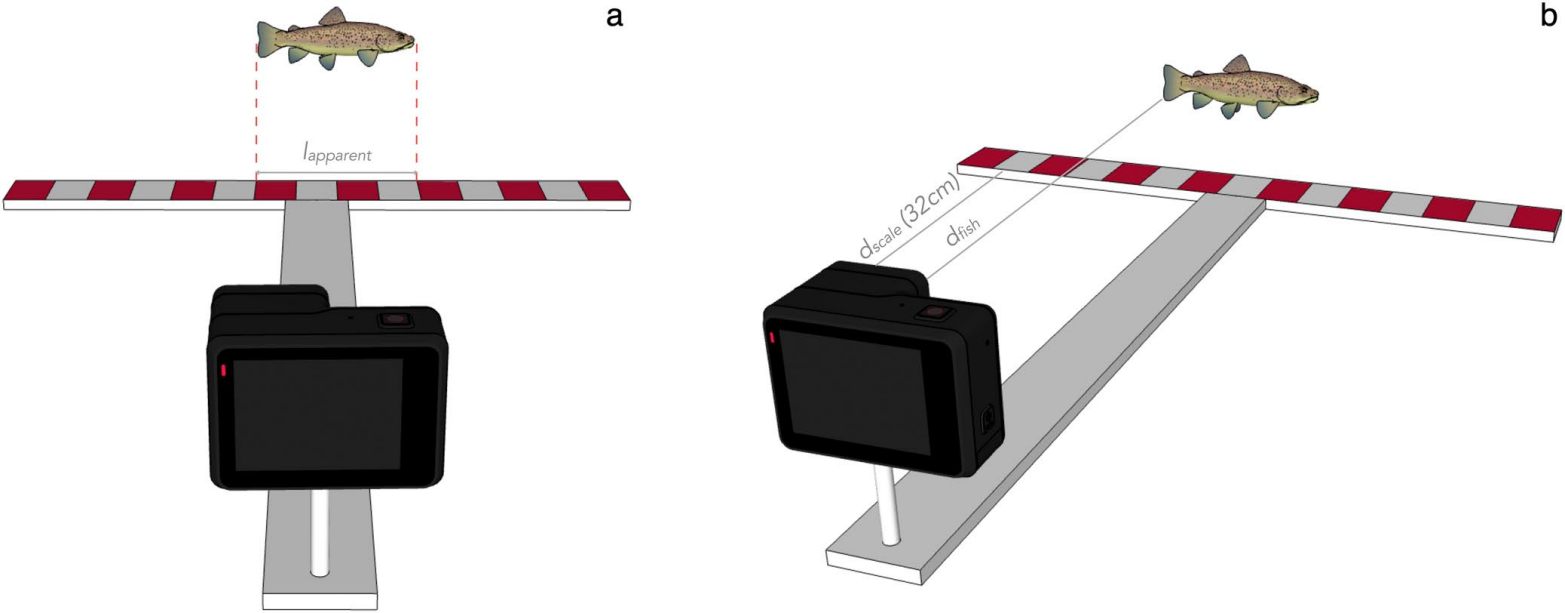
3D representation of the T‐shaped RUV frame showing the estimated measures used to calculate fish length. The 3D model was created with SketchUp (www.sketchup.com).

To estimate the total length (TL) of each fish observed in the footage, we first measured its apparent length ‘*l*_*apparent*_’ using the reference scale (graduated ruler) mounted on the T‐shaped RUV frame. The fish was measured when perpendicular to the camera's field of view, and its projected length was compared to the scale in the same frame (Figure 2a). Salmonids in the surveyed pool were very mobile, and there was at least one frame where each fish was perpendicular to the camera. The next step was to estimate the distance between the fish and the camera, referred to as ‘*d*_*fish*_’. Since the length of the longitudinal bar (32 cm, referred as ‘*d*_*scale*_’) is known, we used this as a reference in the footage, allowing us to estimate how far the fish was from the camera (Figure 2b). This step was relatively easy and accurate, as the pools were relatively small. Following the *d*_*fish*_ estimation, we calculated an estimated fish TL. Specifically, since the apparent size of an object in an image decreases inversely to increasing distance from the camera, the adjusted length estimate of the fish was calculated using the following proportion:

$$\mathrm{TL}=l_{\text{apparent}}\times \left(\frac{d_{\text{fish}}}{d_{\text{scale}}}\right)$$

where *l*_*apparent*_ is the fish's measured length relative to the scale, *d*_*fish*_ is the estimated distance from the camera to the fish, and *d*_*scale*_ is the known distance (32 cm) from the camera to the reference ruler. This correction ensures that the final fish total length estimate accounts for distance‐related distortion in the image.

## 3 Discussion

Freshwater fish play a crucial role in ecosystem function and trophic dynamics in alpine stream systems. There is growing concern that introduced non‐native species risk displacing endemic species (Polgar et al. 2022). Improving our understanding of stream community diversity and abundance, habitat use, fish movement and behaviour in alpine and montane environments (characterised by environmental variability and hydrological isolation) is essential for effective ecosystem conservation.

**FIGURE 3:**
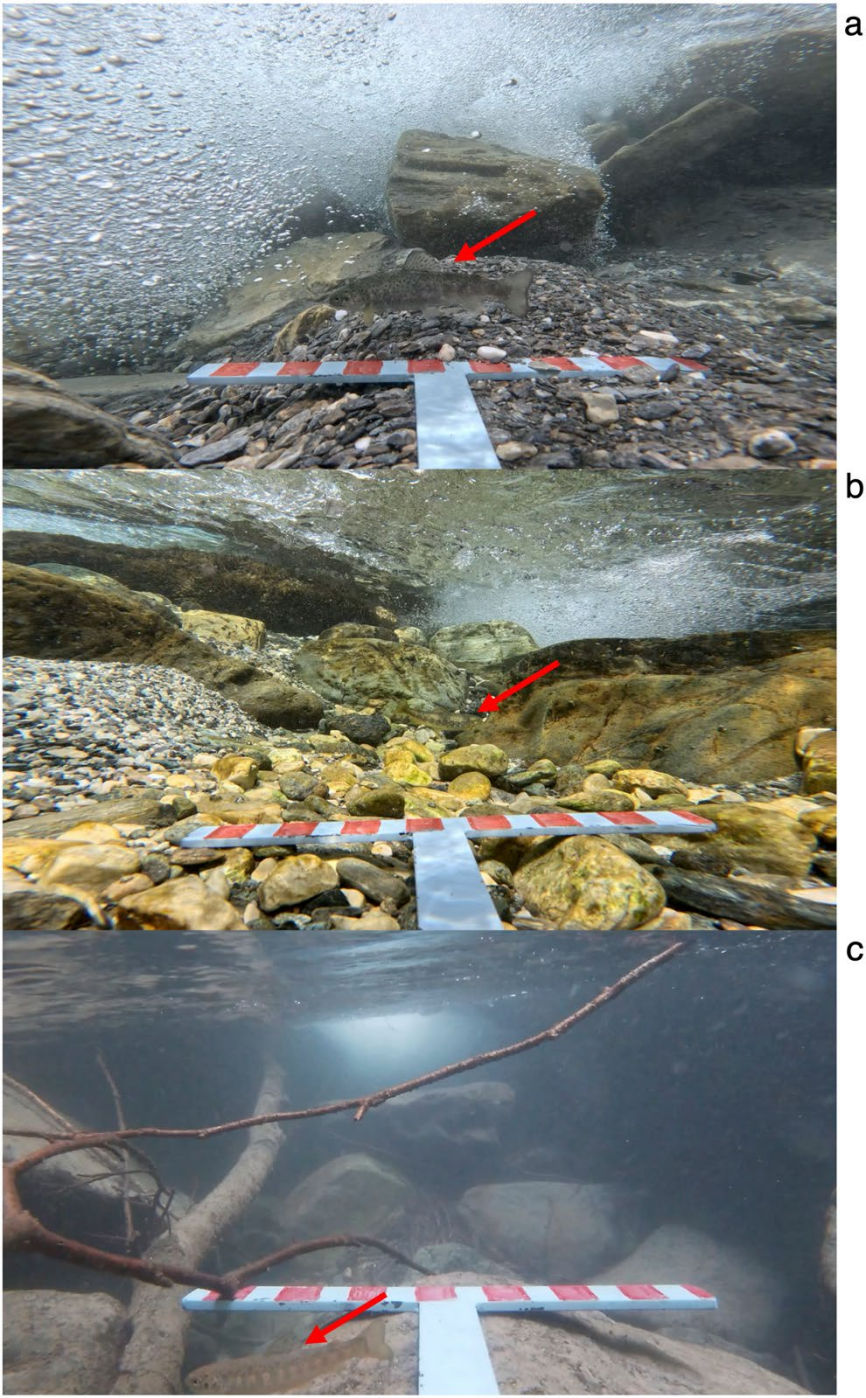
The T‐shaped RUV was effective in capturing salmonid fish in each alpine stream trialed, *Varaita di Sustra* (a), *Rio Bulè* (b) and *Rio Alpetto* (c). Estimated fish TL (total length) in (a) is 18 cm estimated, (b) 17.14 cm and (c) 6.86 cm.

**FIGURE 4:**
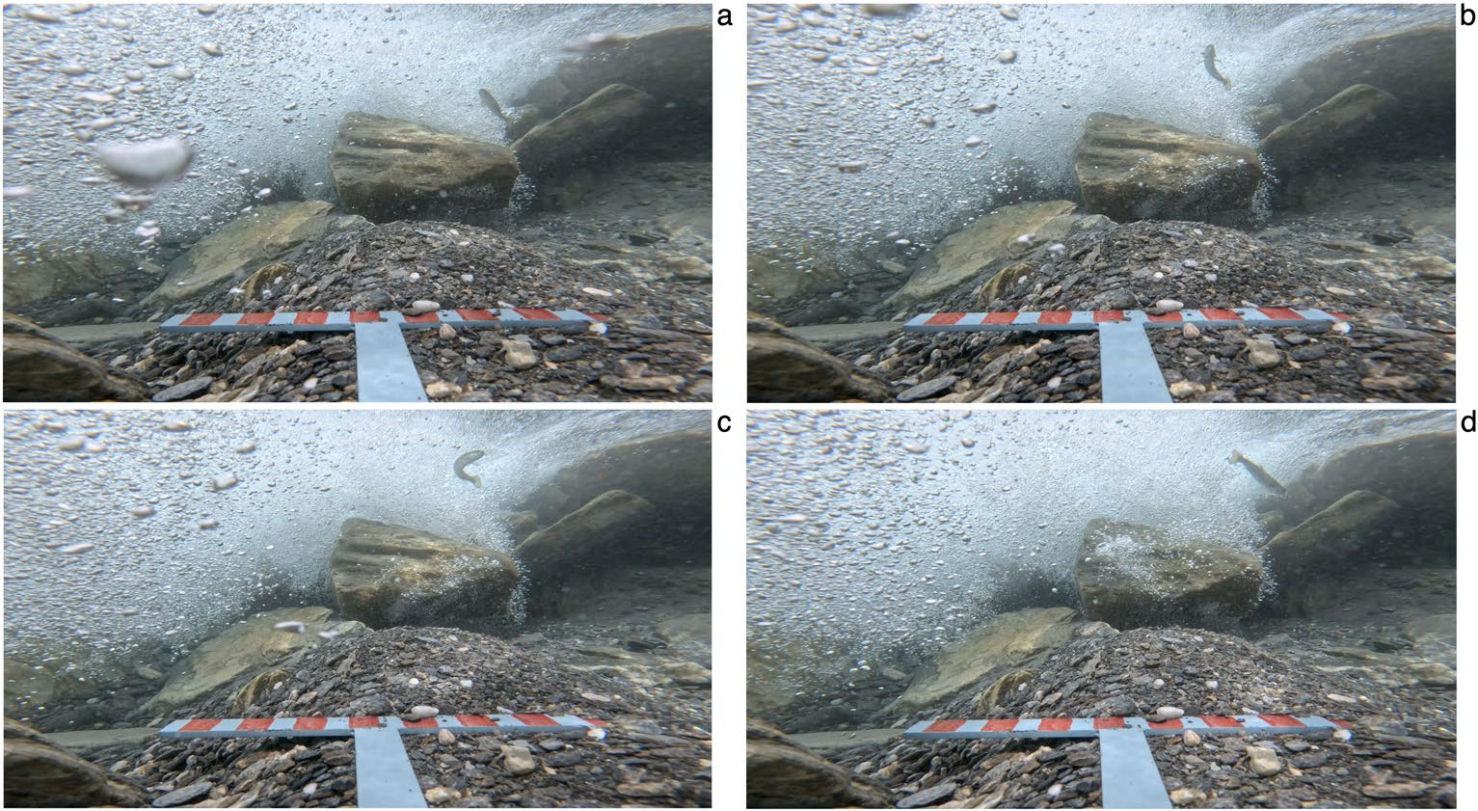
RUV can help analyse feeding behaviour. (a) Salmonid ascending the water column to feed, (b) feeding on a suspended particle, (c and d) and returning to the hideout.

The T‐shaped RUV tested here has proven effectiveness in recording size and abundance estimates of salmonids in Italian alpine streams characterised by high altitude and fast flow (Figure 3). The integrated measuring scales provided consistent size estimates across all observations, improving post‐survey analysis and minimising measurement bias. Beyond simple size and abundance data, the RUV configuration also allows for the observation of natural behaviours (e.g., Figure 4, a salmonid is recorded actively feeding in the water column). This demonstrates the potential of the system not only for fish detection and measurement, but also for observation and analysis of behavioural patterns.

However, the quality of the footage is highly dependent on environmental conditions, and interference from strong currents and air bubbles can significantly limit the interpretability of the recordings. Two main challenges emerged during the trial. The first was to design and construct metal frames that were light enough to be transported on foot over 2‐h periods to reach isolated sites, but were heavy enough to be negatively buoyant (counteract the displacement of the camera) and remain stable on the stream bed without being moved by strong currents. The use of a steel frame weighing ~1 kg proved to be a good compromise. The second challenge was the placement of the system in the stream to avoid air bubbles caused by rapidly cascading water. When placed in unsuitable locations, video imagery could be completely obscured by air bubbles, making the footage unusable for both identification and analysis. This problem was particularly evident during the first tests but became less frequent as the operators developed better positioning strategies.

Time investment for each video sample amounted to ~45 min: filming within a stream pool for 20 min, ~3 min for placement and retrieval, and ~20 min for post‐field analysis/review. Reviewing the footage at double speed was also tested, which successfully reduced viewing time while permitting fish identification and checking for successful deployment; however, this approach may not be suitable for detailed behavioral analysis. Measuring the length of fish from the videos took, on average, about 2 min per individual.

Overall, we believe that the system presented here can be used effectively in waters where traditional visual census methods (e.g., Frehse et al. 2020) are not feasible due to shallow depths, as well as in situations where it represents a better option than electrofishing surveys. We particularly recommend our method for high‐altitude and difficult‐to‐reach locations, as well as for surveys where inexpensive and easy‐to‐use equipment is essential.

## Author Contributions

**Paolo Cappa:** conceptualization (equal), data curation (equal), formal analysis (equal), funding acquisition (equal), investigation (equal), methodology (equal), project administration (equal), resources (equal), software (equal), supervision (equal), validation (equal), visualization (equal), writing – original draft (equal), writing – review and editing (equal). **Matt J. Nimbs:** supervision (equal), writing – original draft (equal), writing – review and editing (equal).

## Conflicts of Interest

The authors declare no conflicts of interest.

## Data Availability

Data used in this study may be obtained at https://zenodo.org/records/15868936.
